# Continuous Long-Term Assessment of Heart Rate Variability in Adults with Cyanotic Congenital Heart Disease after Surgical Repair

**DOI:** 10.3390/jcm13072062

**Published:** 2024-04-02

**Authors:** Felix Pieringer, Mathieu N. Suleiman, Ann-Sophie Kaemmerer-Suleiman, Oliver Dewald, Annika Freiberger, Michael Huntgeburth, Nicole Nagdyman, Rhoia Neidenbach, Fabian von Scheidt, Harald Kaemmerer, Peter Ewert, Michael Weyand, Sebastian Freilinger, Frank Harig

**Affiliations:** 1 Department of Cardiac Surgery, University Hospital Erlangen, Friedrich-Alexander-University Erlangen-Nürnberg, 91054 Erlangen, Germany; 2 International Center for Adults with Congenital Heart Disease, Department of Congenital Heart Disease and Pediatric Cardiology, German Heart Center Munich, Technical University Munich, 80636 Munich, Germany; 3 Department of Sport and Health Sciences, Technical University Munich, 80992 Munich, Germany

**Keywords:** adults with congenital heart disease, ACHD, cyanotic congenital heart defects, heart rate variability, complete transposition of the great arteries, atrial switch, arterial switch, cardiac surgery, Rastelli operation

## Abstract

**Background:** Heart rate variability (HRV) is an established, non-invasive parameter for the assessment of cardiac autonomic nervous activity and the health status in general cardiology. However, there are few studies on HRV in adults with congenital heart defects (CHDs). The aim of the present study was to evaluate the use of long-term continuous HRV measurement for the assessment of global health status in adults with cyanotic CHD. **Methods:** This prospective study included 45 adults (40% female, mean age = 35.2 ± 9.2 [range: 19–58] years) after cardiac surgical repair. HRV parameters were calculated from continuous 24 h measurements using a Bittium Faros 180 sensor (Bittium Corp., Oulu, Finland). **Results:** Postoperative patients with transposition of the great arteries (TGA) (n = 18) achieved significantly higher values of standard deviation of NN intervals (SDNN) (175.4 ± 59.9 ms vs. 133.5 ± 40.6 ms; *p* = 0.013) compared with patients with other conotruncal anomalies (n = 22). Comparing patients with TGA after a Senning–Brom or Mustard operation (n = 13) with all other heart surgery patients (n = 32), significantly higher HRV parameters were found after atrial switch (root mean square of successive RR interval differences: 53.6 ± 20.7 ms vs. 38.4 ± 18.3 ms; *p* = 0.019; SDNN: 183.5 ± 58.4 ms vs. 136.3 ± 45.3 ms; *p* = 0.006). A higher SDNN was also measured after Senning–Brom or Mustard operations than after a Rastelli operations (n = 2) (SDNN: 183.5 ± 58.4 ms vs. 84.5 ± 5.2 ms; *p* = 0.037). When comparing atrial switch operations (n = 3) with Rastelli operations, the SDNN value was significantly shorter in the Rastelli group (*p* = 0.004). **Conclusions:** Our results suggest that continuous HRV monitoring may serve as a marker of cardiac autonomic dysfunction in adults with cyanotic CHD after surgical repair. Impaired cardiac autonomic nervous activity may be associated with an increased risk of adverse reactions in patients with repaired CHD. Therefore, a longitudinal assessment of HRV patterns and trends may provide a deeper insight into dynamic changes in their autonomic regulation and disease progression, lifestyle changes, or treatments. As each person has individual variability in heart rate, HRV may be useful in assessing intra-individual disease progression and may help to improve personalized medicine. Further studies are needed to better understand the underlying mechanisms and to explore the full potential of HRV analysis to optimize medical care for ACHDs.

## 1. Introduction

Congenital heart defects (CHDs) affect approximately 1 in every 100 children born. Due to tremendous advances in medical care, more than 95% of those affected now survive into adulthood [1]. According to current estimates, there are more than 50 million people living with CHDs in the world today [2]. Over the next few decades, this number will continue to increase [3].

All patients with CHDs, whether treated or untreated, have chronic heart disease. The major long-term sequelae and complications include heart disease-specific residua, sequelae, and comorbidities such as heart failure, arrhythmias, pulmonary vascular disease, infective endocarditis, and aortopathies [4,5,6]. CHDs can also lead to acquired non-cardiac comorbidities such as organ disease and/or mental health conditions such as depression, anxiety, and post-traumatic stress disorders [7]. This poses a significant challenge for the entire healthcare system, as lifelong follow-up care is essential [8].

A wide range of different examination methods is required to record and assess the above-mentioned residua and comorbidities, including imaging techniques such as echocardiography, X-ray, magnetic resonance imaging (MRI), and computed tomography (CT). Electrocardiogram (ECG), long-term ECG, telemetry, and cardiopulmonary exercise tests are also of particular importance [9,10]. Another technique is continuous heart rate variability (HRV) measurement [11,12]. HRV includes a number of parameters that reflect the balance of the autonomic nervous system and provide information about the state of health and physiological adaptability of the body [13,14].

HRV has been used for several years as a non-invasive measure of patient health status, particularly in cardiology, sports medicine, occupational medicine, and psychosomatic medicine. However, HRV measurement has not been widely used in the adult population with CHDs.

The aim of the present study was to evaluate the use of long-term continuous HRV measurement for the assessment of global health status in adults with different types of cyanotic CHD. We hypothesized that HRV may help provide deeper insight into the dynamic changes in autonomic regulation, allowing better assessment of disease progression and identifying patients at risk for disease progression.

## 2. Materials and Methods

This joint project of the Department of Cardiac Surgery, Friedrich-Alexander-University Erlangen-Nürnberg, Erlangen, Germany, and the Department of Congenital Heart Disease and Pediatric Cardiology, German Heart Center Munich, Technical University Munich, Munich, Germany, included 45 patients with different types of cyanotic CHDs. All patients in this study were prospectively enrolled from July 2019 to December 2021 in the order of presentation in the outpatient clinic, and no prior selection for inclusion was performed.

Inclusion criteria for this study were a minimum age of 18 years, a diagnosis of cyanotic CHD, previous cardiac surgery, and a fully completed informed consent form. Individuals who met the inclusion criteria were informed about the study during the cardiac evaluation and asked to sign an informed consent document if they wished to participate. Patients with cognitive impairment or language barriers that prevented understanding of the study were excluded. Patients who withdrew from the study, failed to fill out the informed consent form, or incompletely filled out the informed consent form were excluded as well. Individuals with an implanted cardiac pacemaker were also excluded. Further exclusion criteria were HRV measurements of less than 20 h duration or insufficient recording quality due to artifacts.

After the cardiologic examination by a specialist in adults with CHD (ACHDs), the Bittium Faros 180 sensor was placed on the subjects at the level of the sternum using a bipolar electrode, with instructions to wear it for 24 h for the collection of HRV parameters.

HRV parameters, such as standard deviation of NN intervals (SDNN) and root mean square of successive RR interval differences (RMSSD), were determined at a sampling rate of 1000 Hz. At the end of the recording period, the device was removed and returned to the clinic where the data were read and analyzed.

CHD diagnosis was reviewed and confirmed by experienced ACHD-specialist cardiologists and cardiac surgeons. A specially designed documentation form was completed for each patient based on the patient’s current medical records. The form included information on medical history, type of leading heart defect, Perloff functional class, medication, and sociodemographic characteristics. The patients’ N-terminal pro-brain natriuretic peptide (NT-proBNP) levels were also included if they were obtained on the day of HRV measurement.

Finally, the recorded data were erased from the sensor, and the device was cleaned and recharged until reuse.

### 2.1. Ethics Approval and Consent to Participate

All subjects enrolled signed an informed consent form. Patients’ medical care and support were not affected by their participation in this study. The study was approved by the Ethics Committees of the Technical University of Munich and the Friedrich-Alexander University of Erlangen-Nuremberg (reference number: 158/19 S/179_21 Bc; approval date: 25 October 2019). To ensure data security, pseudonyms consisting of numbers and letters were created and patient records were stored separately from the collected data at the German Heart Center Munich.

### 2.2. Systematic Literature Review Search Strategy

We followed the Preferred Reporting Items for Systematic Reviews and Meta-Analyses (PRISMA) guidelines to conduct this systematic review. The following databases were systematically searched: PubMed and SCOPUS using the search terms ((Heart-rate variability) OR (HRV)) AND (adults with cyanotic congenital heart disease). The search strategy was conducted in accordance with the PRISMA-S extension of the PRISMA statement for reporting literature searches in systematic reviews.

We included all articles published in English prior to July 2023. All included articles were identified by two authors (MS and ASK). Afterwards, the full texts of all selected studies were reviewed. No study registries or other online resources were searched. No date, language, or study design filters were used. No additional studies were sought by contacting authors.

Study Quality Assessment: The quality of the included studies was evaluated by MS and ASK.

Data Extraction: Studies were considered eligible for inclusion if (1) they addressed cyanotic adult CHD, (2) each patient in the study underwent an evaluation of heart rate, and (3) they were published in English.

Conversely, studies were excluded if they (1) were duplicate publications or (2) did not fulfill the inclusion criteria.

All original searches were conducted on 20 July 2023.

### 2.3. Statistical Analysis

Data from the Bittium Faros 180 sensor were transferred to the CardioScope program version 1.3.229 (SMART Medical, Moreton in Marsh, Gloucestershire, UK) and stored pseudonymously. The relevant HRV parameters were statistically analyzed using SPSS version 26.0.0.0 (IBM Inc., Armonk, New York, NY, USA).

For quantitative variables, mean ± standard deviation and/or median [min; max] were reported, as appropriate. Shapiro–Wilk tests were used to test for normal distribution. Group differences were analyzed using standard inferential statistical procedures (*t*-test for normal distribution and Mann–Whitney as a nonparametric alternative).

To test for a relationship between the HRV parameters RMSSD and SDNN and the nominal variables, the eta coefficient and the eta squared coefficient were calculated. Correlation with the other metric variables was tested with the Spearman Rho correlation because of the lack of normal distribution for three of the four variables. *p* < 0.05 was considered statistically significant.

## 3. Results

### 3.1. Systematic Literature Review Search

In total, five unique citations were identified in SCOPUS and ten in PubMed after an initial search. Of these 15, 13 were initially excluded after screening mainly because their titles were unrelated to the current study. The remaining two studies were reviewed but did not match the discussed topic.

Additional articles were manually identified by screening the cited references. In total, 57 references were checked, and all were excluded due to not matching the discussed topic.

After an extensive literature search under the above keywords, no articles were found that addressed the topic we are describing. Therefore, the present study is likely the first in the literature to specifically describe HRV in adult patients with cyanotic CHDs.

### 3.2. Demographic and Clinical Data

A total of 45 adults with primary cyanotic CHDs met the inclusion criteria. These were complete transposition of the great arteries (TGA) (n = 18), tetralogy of Fallot (TOF) (n = 11), double outlet right ventricle (DORV, Fallot type, n = 5), double inlet ventricle (DIV) (n = 2), truncus arteriosus communis (TAC) (n = 2), pulmonary atresia with ventricular septal defect (PA-VSD) (n = 2), tricuspid atresia (TrA) (n = 1), pulmonary valve agenesia (n = 2), univentricular heart (UVH) (n = 1), and pulmonary atresia with intact septum (PA-IVS) (n = 1) (Table 1 and Table 2).

All patients had undergone cardiac surgery for CHD (Table 2). Age at study inclusion ranged from 19 to 58 years, with a mean age of 35.3 ± 10.2 years for women (n = 18, 40%) and 35.2 ± 8.7 years for men (n = 27, 60%).

At follow-up, 42/45 patients (93.3%) were in Perloff functional class I or II; only 3/45 (6.7%) were in functional class III or IV. NT-proBNP was found in a total of 35/45 (77.8%) patients. The median was 195.0 ng/L (25th percentile: 71.4 ng/L; 75th percentile: 382.0 ng/L).

With regard to history, 10/45 (22.2%) patients had a history of atrial arrhythmias requiring therapy (TGA = 6, TOF = 1, DIV = 1, PA with VSD = 1, TrA = 1), and 3/45 (6.7%) patients had ventricular arrhythmias (TGA = 1, TOF = 1, DORV = 1) that had been treated. Antiarrhythmic medication was taken by 17/45 (37.8%) patients: beta-blockers (n = 17; TGA = 8, DORV = 3, TOF = 2, DIV = 1, PA with VSD = 1, TAC = 1, TrA = 1), amiodarone (n = 0), or verapamil (n = 0).

A total of 13/45 (28.9%) subjects had aortopathy (TGA = 3, TOF = 3, DORV = 3, pulmonary valve agenesis = 1, PA with intact ventricular septum = 1, TAC = 1, TrA = 1). Severe pulmonary hypertension was present in 4/45 (8.9%) patients.

Relevant psychological distress was evident in 8/45 (17.8%) patients (TGA = 2, TOF = 1, DORV = 1, PA with VSD = 1, pulmonary valve agenesis = 1, TrA = 1, univentricular heart = 1).

### 3.3. Heart Rate Variability

**Figure 1:**
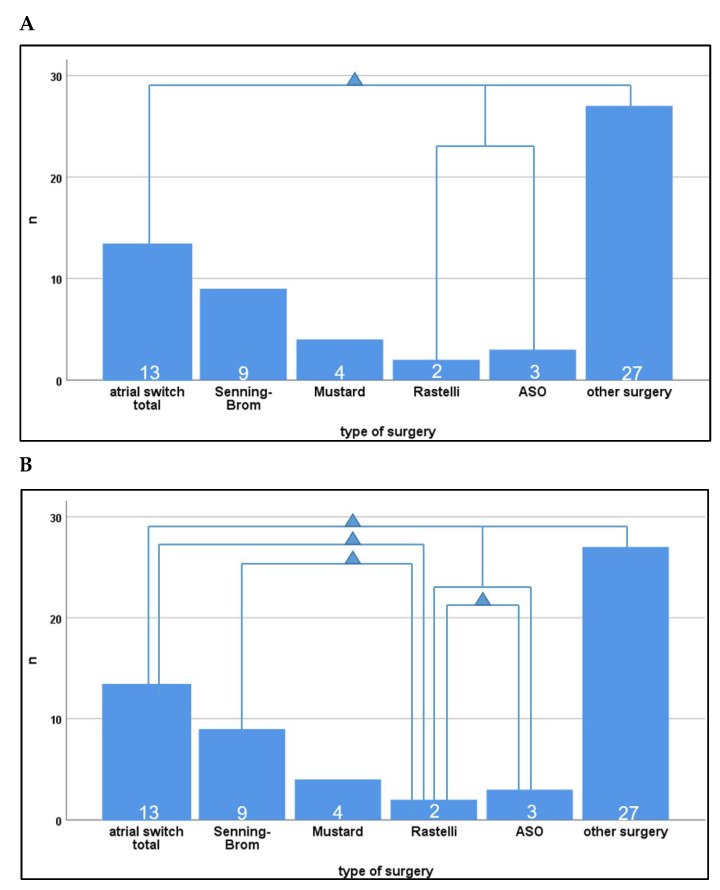
Comparison of RMSSD and SDNN values for patients after six categories of surgery. Significant HRV differences between the different types of surgery are symbolized with a triangle. (**A**): differences in the RMSSD value; (**B**): differences in the SDNN value. RMSSD = root mean square of successive RR interval differences; SDNN = standard deviation of NN intervals; HRV = heart rate variability; ASO = arterial switch operation; n = number of patients.

In this study, RMSSD and SDNN were the assessed HRV parameters (Figure 1).

The mean recording time was 25.2 ± 2.1 h, ranging from 21.0 to 31.3 h. The difference in recording time was due to technical problems or patient application problems.

In the entire group, the measured RMSSD value was 42.8 ± 20.0 ms (median 37.7 ms) and the SDNN value was 149.9 ± 53.3 ms (median 139.6 ms).

By gender, the RMSSD and the SDNN were insignificantly lower in women than in men (RMSSD of 41.0 ± 12.4 ms vs. 44.0 ± 24.0 ms; SDNN of 146.6 ± 53.1 ms vs. 152.1 ± 54.4 ms).

Comparison of patients with TGA (n = 18) to patients with other conotruncal anomalies (n = 22) or univentricular hearts (n = 4) revealed differences depending on the underlying disease and the type of cardiac surgery performed (Figure 1, Table 3).

Patients with TGA (n = 18) achieved significantly higher SDNN values (175.4 ± 59.9 ms vs. 133.5 ± 40.6 ms; *p* = 0.013) compared with patients with other conotruncal anomalies (n = 22). Regarding the RMSSD value, this comparison only showed a trend towards increased values (49.1 ± 19.7 ms vs. 38.7 ± 20.3 ms; *p* = 0.069). Compared with patients with functionally univentricular hearts (n = 4), no differences were measured in patients with TGA (RMSSD: 40.0 ± 19.6 ms vs. 49.1 ± 19.7 ms; *p* = 0.268; SDNN: 125.5 ± 55.8 ms vs. 175.4 ± 59.9 ms; *p* = 0.144). No significant differences were measured between patients with other conotruncal anomalies and patients with functionally univentricular hearts (RMSSD: 38.7 ± 20.3 ms vs. 40.0 ± 19.6 ms; *p* = 0.864; SDNN: 133.5 ± 40.6 ms vs. 125.5 ± 55.8 ms; *p* = 0.732).

In the present study, of 18 patients with TGA, 13 had undergone an atrial switch operation (ASO) after a Senning–Brom or Mustard operation (Senning–Brom, n = 9; Mustard, n = 4), 2 had undergone a Rastelli operation, and 3 had undergone an ASO.

Comparing patients with TGA after Senning–Brom or Mustard operation (n = 13) with all other surgically treated patients with cyanotic heart defects (n = 32), significantly higher HRV parameters were found after atrial switch (RMSSD: 53.6 ± 20.7 ms vs. 38.4 ± 18.3 ms; *p* = 0.019; SDNN: 183.5 ± 58.4 ms vs. 136.3 ± 45.3 ms; *p* = 0.006).

No significant differences were found between the Senning–Brom (n = 9) and the Mustard (n = 4) techniques in the TGA patients.

A higher SDNN was also measured after Senning–Brom or Mustard operations compared with patients after Rastelli operation (n = 2) (SDNN: 183.5 ± 58.4 ms vs. 84.5 ± 5.2 ms; *p* = 0.037).

No differences were found after Senning–Brom or Mustard operations compared with ASO.

However, comparing ASO with the Rastelli operation, the SDNN value was significantly shorter in the Rastelli group (*p* = 0.004).

Comorbidities in the form of atrial arrhythmias are also relevant in TGA patients. In the entire group, TGA patients with a history of atrial arrhythmia requiring therapy (n = 6) had significantly lower SDNN values (*p* = 0.028) compared with TGA patients without this arrhythmia (n = 12).

## 4. Discussion

Data on HRV in CHD patients are limited. The present study provides the first comprehensive, prospective data on HRV in a moderately large (in the context of a rare condition) population of adults with cyanotic CHDs and cardiac or non-cardiac comorbidities. While previous studies of HRV in CHD patients have almost exclusively used older techniques that allow only short-term HRV measurements, in the present study, HRV was recorded over 24 h.

As studies in cardiology, sports medicine, occupational medicine, and psychosomatics have shown, analysis of HRV provides valuable insights into the dynamic balance between the sympathetic and parasympathetic branches of the autonomic nervous system and thus allows conclusions on the state of health and physiological adaptation [13,15] (Table 4). In this regard, a large variation between heartbeats indicates a high-performance status, and a lack of variation indicates impaired autonomic control, reduced adaptability to internal and external influences, a stressed organism, or disease [13]. Altered HRV parameters can also be used for prognosis and risk stratification, as they may precede the onset of arrhythmias, organ damage, the development of heart failure, or sudden death [16,17] (Table 5).

As up to 95% of patients with CHDs now reach adulthood in the developed world, most with significant residua and sequelae as well as cardiac and non-cardiac comorbidities, it is important to identify appropriate prognostic and risk parameters to facilitate counseling to make lifestyle, exercise, and treatment safer and more effective [18]. This is especially true for complex CHDs after previous surgical or interventional procedures. As these may influence autonomic cardiovascular regulation, HRV assessment is an important diagnostic tool.

Small studies in pediatric cardiology have shown that HRV has the potential to predict adverse outcomes in patients with CHDs, and that HRV assessment may identify patients who require more intensive follow-up and targeted risk reduction measures [17].

The largest study of HRV and autonomic nervous system function in CHD patients included 258 children [19]. Unfortunately, this study did not differentiate between types of CHD, age groups, chosen procedures (e.g., transcutaneous or via sternotomy/thoracotomy), untreated or postoperative course, pressure and/or volume overload, presence of heart failure or pulmonary vascular disease, or different long-term courses affecting autonomic nervous system function. Similar drawbacks exist in other small studies on this topic [14,20,21,22,23,24].

In contrast, compared with the existing pediatric cardiology studies, the present study included 45 adults (mean age 35.7 ± 9.6 years) after operative repair, 40% (n = 18) of whom were women. With the exception of a single patient with pulmonary atresia and intact ventricular septum, the group included patients after repair of various forms of conotruncal anomalies, including complete TGA, tetralogy of Fallot, pulmonary atresia with ventricular septal defect, DORV, truncus arteriosus communis, and univentricular heart. Most patients were able to function well in daily life, with 42 patients (93.3%) in Perloff functional class I or II and a mean NT-pro-BNP level of 423.5 ± 836.5 ng/L. Relevant comorbidities were found in 13 patients in the form of arrhythmias (n = 13, 28.9%), pulmonary hypertension (n = 4, 8.9%), aortopathy (n = 13, 28.9%), or mental illness (n = 8, 17.8%).

Within the group of conotruncal defects, the group of patients with TGA represents an important subgroup. In the literature, descriptions of HRV are limited in patients with TGA who have undergone a Senning–Brom, Mustard, or Rastelli operation, or an atrial switch procedure.

In small cohorts, altered HRV parameters have been inconsistently described, also suggesting dysregulation of the autonomic nervous system with decreased parasympathetic tone and increased sympathetic activity [14]. Underlying causes include impaired autonomic regulation due to structural abnormalities, surgical technique, cardiac scarring, postoperative changes, residual and secondary cardiovascular disease, and precipitating factors such as the degree and duration of preoperative cyanosis.

In 29 adults with TGA after Mustard or Senning–Brom operations, Zandstra et al. found decreased HRV parameters in long-term HRV measurements. However, in this study, the available 24 h ECGs were analyzed retrospectively [25].

In contrast to atrial switch surgery, during the arterial switch operation for TGA, the aorta and the pulmonary trunk are separated at their base and placed in the anatomically correct position [26]. This may damage sympathetic nervous structures along the origin of the great arteries, affecting autonomic regulation [27]. Such changes in autonomic control may be reversible, especially if the surgery is performed early (before 55 days of life) [28].

In the present study, out of 18 patients with TGA, 13 had Senning–Brom (n = 9) or Mustard (n = 4) operations, 2 had a Rastelli operation, and 3 had an ASO. When analyzing the HRV parameters, post-Senning–Brom operation patients (n = 9) did not differ from post-Mustard operation patients (n = 4).

However, comparing post-Senning–Brom operation patients and post-Mustard operation patients with patients after Rastelli surgery (n = 2), there were significant differences in the SDNN value (*p* = 0.037); it was significantly shorter after a Rastelli operation than after Senning–Brom or Mustard operations.

No differences were found between post-ASO and post-atrial switch surgery patients, but significant differences were found between post-ASO and post-Rastelli operation patients, in whom the mean SDNN value was significantly shorter (*p* = 0.004). Importantly, TGA patients with a history of atrial arrhythmia requiring therapy (n = 6) had significantly lower SDNN values (*p* = 0.028) than TGA patients without arrhythmia (n = 12).

Another important subgroup of conotruncal defects is Fallot’s tetralogy. In the literature, HRV-related findings in patients with tetralogy of Fallot are not consistent. On the one hand, postoperative Fallot patients often show altered HRV parameters compared with patients without CHD, suggesting an imbalance in autonomic regulation, including reduced total HRV, decreased parasympathetic activity, and increased sympathetic modulation [29]. In contrast, Wyller et al. found no significant differences in HRV analysis with respect to autonomic regulation between postoperative Fallot patients and controls [30]. Nevertheless, a causal relationship is assumed with the type of surgery chosen, the extent and duration of preoperative cyanosis, and disturbed parasympathetic tone (e.g., in the context of right ventricular overloading and/or altered pulmonary blood flow) or due to scarring after atriotomy or ventriculotomy [31].

In the current study of ACHDs, significantly higher HRV parameters were observed after Senning–Brom or Mustard operations, compared with the 32 postoperative patients with cyanotic CHD, including those with tetralogy of Fallot and related anomalies such as pulmonary atresia (RMSSD: 53.6 ± 20.7 ms vs. 38.4 ± 18.3 ms; *p* = 0.019; SDNN: 183.5 ± 58.4 ms vs. 136.3 ± 45.3 ms; *p* = 0.006).

## 5. Limitations of the Study

The present study has several limitations. One limitation may arise from the relatively small and heterogeneous cohort of patients recruited. The number of patients was limited due to the rarity of the disease, which may restrict the interpretation of the data.

This study was performed in a tertiary care center for adults with CHD. Therefore, the sample of patients does not represent the typical population with CHD seen by general practitioners or cardiologists. The prevalence of high-risk patients in our institution is likely to be higher than that in regional hospitals or in regular cardiology departments. Therefore, the patient group might be biased towards more symptomatic patients.

On the other hand, HRV parameters may be less favorable in patients who are not treated in an ACHD center with experienced specialists.

In addition, HRV parameters are highly sensitive to external factors, and norms for the analysis of HRV parameters and prognostically significant impaired values of these parameters have not yet been developed in ACHDs.

We used time domain parameters of HRV. Spectral analysis or other techniques could provide a more detailed analysis of HRV and further increase the prognostic power of HRV parameters. However, the prognostic value of time domain HRV parameters is well established in non-congenital heart disease cohorts.

Finally, further studies are needed to determine the value of HRV for long-term prognosis in patients with CHDs.

## 6. Conclusions and Future Perspectives

HRV is a dynamic parameter that reflects the complex interaction between the sympathetic and parasympathetic branches of the autonomic nervous system and cardiovascular health.

Reduced HRV after repair of complex cyanotic heart defects may be associated with adverse cardiovascular outcomes, including arrhythmias, impaired exercise tolerance, and increased risk of mortality. Therefore, the present findings appear to be of clinical importance for therapy management and prognostic and risk stratification.

The advantage of continuous recording of HRV, which was applied in the current study, is the uninterrupted monitoring of HRV over longer periods of time, providing a more comprehensive assessment of autonomic function, and a greater accuracy in detecting subtle changes in autonomic activity.

Continuous HRV registration allows longitudinal assessment of HRV patterns and trends, providing deeper insight into dynamic changes in autonomic regulation and identifying effects of disease progression, lifestyle changes, or therapies.

Therefore, continuous HRV registration has the potential to improve personalized medicine. With the advent of wearable devices and smartphone applications, individuals can track their own HRV patterns and thus take an active role in managing their cardiovascular health.

Further study is therefore needed to better understand the underlying mechanisms and explore the full potential of HRV analysis to optimize patient care.

## Figures and Tables

**Table 1:** Characteristics of patients with primary cyanotic congenital heart defects, divided into groups I–IV (I: TGA; II: other conotruncal anomalies; III: univentricular heart; IV: others).

	Group I	Group II	Group III	Group IV
	TGA	Other conotruncal anomalies (DORV, TAC, TOF, PA-VSD, PVA)	Univentricular heart (DIV, TrA, UVH)	Others (PA with intact septum)
	n	n	n	n
	(% within group)	(% within group)	(% within group)	(% within group)
**n**	18 (100)	22 (100)	4 (100)	1 (100)
**Sex female/male**	7 (38,9)/ 11 (61.1)	11 (50)/ 11 (50)	0 (0)/ 4 (100)	0 (0)/ 1 (100)
**Surgical repair**	18 (100)	22 (100)	4 (100)	1 (100)
**Functional class according to Perloff** **I + II/III + IV**	18 (100)/ 0 (0)	20 (90.9)/ 2 (9.1)	3 (75)/ 1 (25)	1 (100)/ 0 (0)
**Cyanosis**	2 (11.1)	1 (4.5)	2 (50)	0 (0)
**Pulmonary hypertension**	1 (5.6)	3 (13.6)	0 (0)	0 (0)
**Aortopathy**	3 (16.7)	8 (36.4)	1 (25)	1 (100)
**Atrial arrhythmias**	6 (33.3)	2 (9.1)	2 (50)	0 (0)
**Ventricular arrhythmias**	1 (5.6)	2 (9.1)	0 (0)	0 (0)
**Drugs** **Beta-blockers** **Amiodarone** **Verapamil**	8 (44.4) 0 (0) 0 (0)	7 (31.8) 0 (0) 0 (0)	2 (50) 0 (0) 0 (0)	0 (0) 0 (0) 0 (0)
**Stroke or TIA**	0 (0)	0 (0)	0 (0)	0 (0)
**Psychiatric disorders**	2 (11.1)	4 (18.2)	2 (40)	0 (0)

DIV = double inlet ventricle; DORV = double outlet right ventricle; PA = pulmonary atresia; PVA = pulmonary valve agenesia; TAC = truncus arteriosus communis; TGA = transposition of the great arteries; TIA = transitory ischemic attack; TOF = tetralogy of Fallot; TrA = tricuspid atresia; UVH = univentricular heart; VSD = ventricular septal defect.

**Table 2:** Overview of study subjects with individual clinical data and HRV parameters.

Type of CHD	Year of First Surgery	Age at First Repair (Years)	Number of Subsequent Cardiac Operations	Interval: Repair to HRV Measurement (Years)	Cardiac Surgery	RMSSD Value (ms)	SDNN Value (ms)
DIV	1983	1	2	37	Atrioseptectomy, aortopulmonary shunt	21.2	62.9
DIV	1999	1	2	21	TCPC	29.2	104.6
DORV	1986	6	0	34	Repair with intraventricular tunnel patch	80.5	190.1
DORV	1994	1	2	26	Kawashima operation with intracavitary tunnel patch	32.5	105.8
DORV	1991	1	7	29	Modified Blalock-Taussig shunt	37.7	124.4
DORV	1983	1	0	37	ASO, VSD patch closure, ASD direct closure	16.3	124.1
DORV	1994	2	2	27	PCPC, pulmonary valve commissurotomy, central aortopulmonary shunt, RPA reconstruction	19.7	114.0
PA + VSD	2001	1	1	20	Unifocalization, RV-PA conduit	36.3	95.3
PA + VSD	1988	1	2	32	Banding of a MAPCA	30.2	87.1
PA intact septum	2006	24	0	14	RV-PA allograft implantation, tricuspid valvuloplasty	30.3	150.8
PVA	2007	7	0	13	RV-RA conduit, VSD patch closure	28.6	102.9
PVA	2003	22	0	17	Allograft implantation, ASD patch closure, PDA closure	28.2	188.6
TAC	1989	1	2	31	Pulmonary homograft, truncal valve repair	16.0	92.0
TAC	1986	1	2	34	Repair with Hancock conduit	51.7	163.6
TGA	1997	1	0	23	ASO	44.9	191.1
TGA	1987	1	0	33	ASO	30.3	188.0
TGA	1998	1	0	22	ASO, VSD direct closure, ASD patch closure	50.5	223.2
TGA	1996	5	2	24	Rastelli operation	22.5	80.8
TGA	1992	16	2	28	Rastelli operation	39.0	88.2
TGA	1983	1	0	37	Mustard operation	22.8	111.4
TGA	1979	6	0	41	Mustard operation	45.2	172.6
TGA	1975	3	1	44	Mustard operation	73.1	278.6
TGA	1979	1	1	41	Mustard operation	37.9	141.4
TGA	1982	1	0	39	Senning–Brom operation	75.7	253.0
TGA	1973	1	0	47	Senning–Brom operation	49.2	222.0
TGA	1980	1	1	39	Senning–Brom operation	69.1	181.5
TGA	1980	1	0	40	Senning–Brom operation	69.4	166.4
TGA	1984	1	0	37	Senning–Brom operation	45.7	160.4
TGA	1992	1	0	28	Senning–Brom operation	26.4	191.0
TGA	1984	2	0	37	Senning–Brom operation	41.2	136.4
TGA	1983	1	0	37	Senning–Brom operation	92.7	273.0
TGA	1991	1	0	29	Senning–Brom operation	48.3	97.3
TOF	1994	1	1	26	TOF repair	55.5	187.5
TOF	1982	5	0	38	TOF repair	33.0	132.5
TOF	1983	2	2	37	TOF repair	29.1	79.4
TOF	1997	2	1	23	TOF repair	50.9	191.5
TOF	1985	1	0	35	TOF repair	52.8	195.1
TOF	1971	9	1	52	TOF repair	99.7	109.4
TOF	1972	5	1	48	TOF repair	33.5	121.5
TOF	1975	5	0	44	TOF repair	17.0	99.6
TOF	2000	2	0	20	TOF repair	33.5	139.0
TOF	1975	1	0	44	TOF repair	34.0	98.8
TOF	1988	1	0	32	TOF repair	35.3	195.4
TrA	1980	3	4	41	Aortopulmonary shunt, PCPC	65.8	139.6
UVH	1985	1	1	35	Fontan operation, CoA repair	43.8	194.8

HRV = heart rate variability; ASD = atrial septal defect; ASO = atrial switch operation (Mustard or Senning–Brom); CHD = congenital heart defect; DIV = double inlet ventricle; DORV = double outlet right ventricle; MAPCA = major aortopulmonary collateral; ms = milliseconds; PA = pulmonary atresia; PCPC = partial cavopulmonary anastomosis; PDA = patent ductus arteriosus; PVA = pulmonary valve agenesia; RPA = right pulmonary artery; RV-PA conduit = conduit from right ventricle to pulmonary artery; TAC = truncus arteriosus communis; TCPC = total cavopulmonary anastomosis; TGA = transposition of the great arteries; TOF = tetralogy of Fallot; TrA = tricuspid atresia; UVH = univentricular heart; VSD = ventricular septal defect.

**Table 3:** HRV parameters depending on the underlying surgical procedure.

			*p*-Value
**TGA vs. other conotruncal anomalies**			
	TGA	Other conotruncal anomalies	
n	18	22	
RMSSD (ms)	49.1 ± 19.7	38.7 ± 20.3	0.069
SDNN (ms)	175.4 ± 59.9	133.5 ± 40.6	**0.013**
**Senning–Brom/Mustard (atrial switch) vs. other surgeries**			
	Senning–Brom/Mustard (atrial switch)	Other surgeries	
n	13	32	
RMSSD (ms)	53.6 ± 20.7	38.4 ± 18.3	**0.019**
SDNN (ms)	183.5 ± 58.4	136.3 ± 45.3	**0.006**
**Senning–Brom vs. Mustard**			
	Senning–Brom	Mustard	
n	9	4	
RMSSD (ms)	57.5 ± 20.5	44.8 ± 21.1	0.260
SDNN (ms)	186.8 ± 55.6	176.0 ± 72.8	0.774
**Senning–Brom/Mustard (atrial switch) vs. Rastelli operation**			
	Senning–Brom/Mustard (atrial switch)	Rastelli operation	
n	13	2	
RMSSD (ms)	53.6 ± 20.7	30.8 ± 11.7	0.089
SDNN (ms)	183.5 ± 58.4	84.5 ± 5.2	**0.037**
**Senning–Brom/Mustard (atrial switch) vs. ASO**			
	Senning–Brom/Mustard (atrial switch)	ASO	
n	13	3	
RMSSD (ms)	53.6 ± 20.7	41.9 ± 10.4	0.521
SDNN (ms)	183.5 ± 58.4	200.8 ± 19.5	0.628
**ASO vs. Rastelli operation**			
	ASO	Rastelli operation	
n	3	2	
RMSSD (ms)	41.9 ± 10.4	30.8 ± 11.7	0.400
SDNN (ms)	200.8 ± 19.5	84.5 ± 5.2	0.004

Significant *p*-values are indicated in bold.

**Table 4:** Physiological basis of heart rate variability (HRV) and how it may be altered by CHDs and various surgical interventions.

Sympathetic nervous system (SNS) activity	The SNS plays a crucial role in regulating heart rate and cardiac contractility. Sympathetic fibers from the thoracic and lumbar regions of the spinal cord innervate the heart, releasing neurotransmitters such as norepinephrine. Activation of the SNS increases heart rate, cardiac contractility, and conduction velocity through the release of norepinephrine, which binds to beta-adrenergic receptors on cardiac myocytes and cardiac pacemaker cells. In congenital heart defects, alterations in cardiac anatomy or surgical interventions can disrupt sympathetic innervation. During corrective surgery, manipulation of cardiac structures and incisions may damage sympathetic nerve fibers, leading to impaired sympathetic modulation of heart rate variability.
Parasympathetic nervous system (PNS) activity	The PNS, primarily mediated by the vagus nerve, has an inhibitory control over heart rate. Vagal fibers release acetylcholine, which binds to muscarinic receptors on cardiac pacemaker cells, slowing heart rate and decreasing conduction velocity. Increased parasympathetic tone leads to higher heart rate variability due to the varying influence of the PNS on heart rate. Decreased parasympathetic tone or vagal withdrawal results in reduced HRV, indicating a shift towards sympathetic dominance and decreased adaptability of the cardiovascular system.
Impact of congenital heart anomalies and cardiac surgery	In patients with CHD, structural abnormalities such as abnormal positioning of great arteries, septal defects, or abnormalities in atrial and ventricular structures can directly impact autonomic innervation. Surgical interventions, including corrective procedures such as arterial or atrial switch operations (e.g., Senning–Brom or Mustard procedures), aid in transposition of the great arteries. Procedures such as Rastelli or operations or Fallot repair may further disrupt autonomic regulation. For example, during operative procedures, sympathetic nerve fibers along the great arteries may be damaged, leading to decreased sympathetic modulation of heart rate variability. Additionally, scar tissue formation from surgical incisions or procedures can alter conduction pathways and disrupt autonomic function, further impacting HRV.
Comorbidities and additional factors	Pre-existing conditions commonly seen in CHDs, such as pulmonary hypertension or arrhythmias, can exacerbate autonomic dysfunction. Pulmonary hypertension, for instance, imposes increased workload on the right ventricle, potentially leading to sympathetic overactivity and reduced parasympathetic tone, which can manifest as altered HRV.

**Table 5:** Insights from different HRV parameters and measurement techniques. Depending on the available system, the following HRV parameters are commonly evaluated to gain a comprehensive understanding of an individual’s autonomic nervous system function and cardiovascular health. It is recommended to perform the HRV tests in series, with each patient ideally representing their own control.

Parameters	Measures/Techniques	Insight
Time domain parameters	SDNN (standard deviation of NN intervals) SDANN (standard deviation of the average NN intervals) RMSSD (root mean square of successive differences)	Insights into overall variability and short-term fluctuations in heart rate.
Frequency domain parameters	Techniques like Fourier analysis or autoregressive modeling. Key frequency domain measures ○ LF (low frequency); ○ HF (high frequency); ○ LF/HF ratio.	These parameters analyze the power spectrum of heart rate variability. Key frequency domain measures reflect the balance between sympathetic and parasympathetic nervous system activity.
Nonlinear parameters	Measures like approximate entropy (ApEn) and sample entropy (SampEn)	To assess the complexity and irregularity of heart rate fluctuations. Additional insights beyond traditional linear analyses.
Time–frequency domain parameters	Parameters such as wavelet analysis or short-time Fourier transform	These parameters combine aspects of both time and frequency domains to capture dynamic changes in heart rate variability over time.

## Data Availability

No new data were created or analyzed in this study. Data sharing is not applicable to this article.
